# Supplementary figures and images for: Arl15 upregulates the TGFβ family signaling by promoting the assembly of the Smad-complex
Source: eLife. 2022 Jul 14;11:e76146. doi: 10.7554/eLife.76146 (PMC9352346; doi:10.7554/eLife.76146)

Figure 1 - source data 1

Fig. 1c

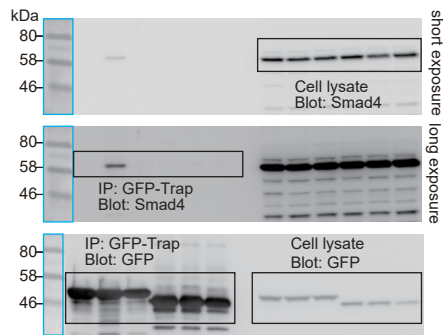

Fig. 1b

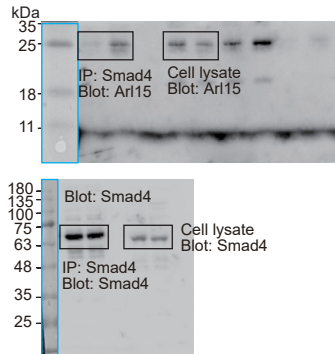

Fig. 1c

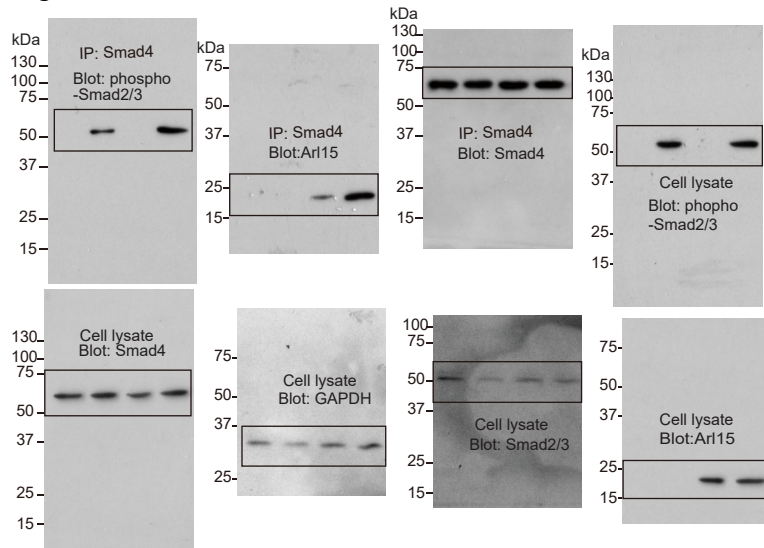

Fig. 1e

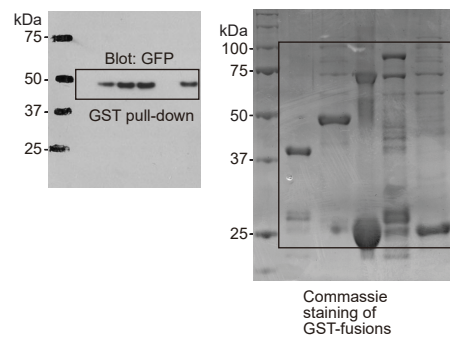

Fig. 1g

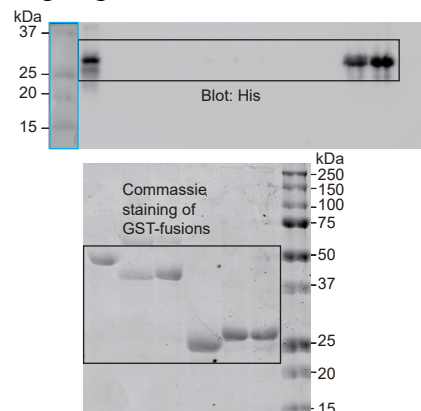

Fig. 1h

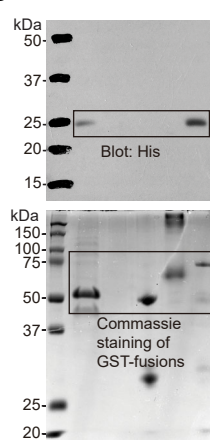

Fig. 1i

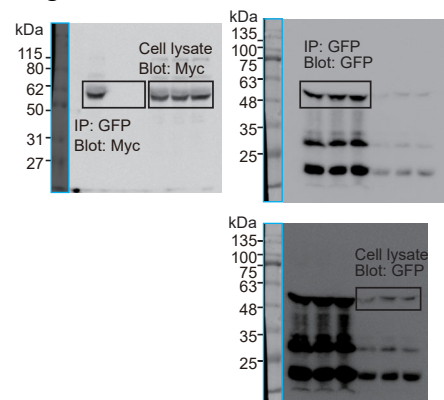

Fig. 1j

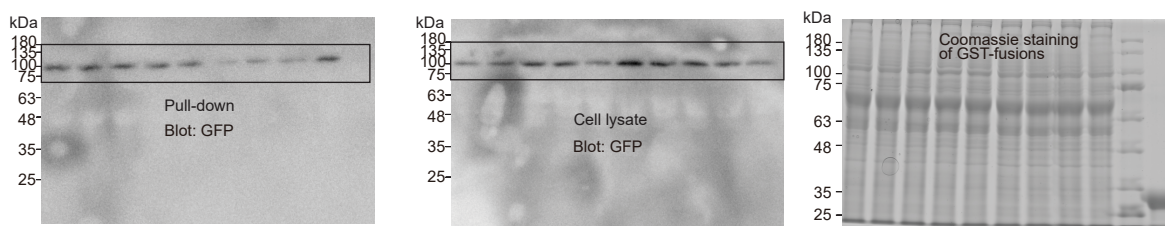

Supplement: Figure 1—source data 1. — Chemiluminescence images are unboxed; black box, the cropped region shown in the corresponding figures; blue box, the white light image of pre-stained molecular weight marker bands (for immunoblots acquired by the CCD camera). Molecular weight (kDa) is labeled in all blots. For immunoblots acquired by film, molecular weight markers were manually traced. [file elife-76146-fig1-data1.pdf]

Figure 1 - source data 2

Figure 1-  
figure supplement1c

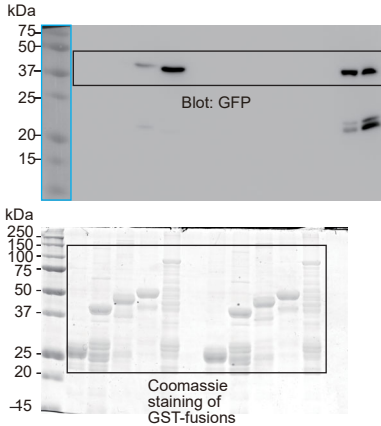

Figure 1-  
figure supplement1d

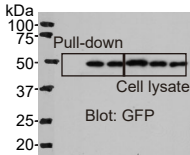

Supplement: Figure 1—figure supplement 1—source data 1. — The organization of the figure is similar to that of Figure 1—source data 1. [file elife-76146-fig1-figsupp1-data1.pdf]

Figure 3 - source data 1

Fig. 3a

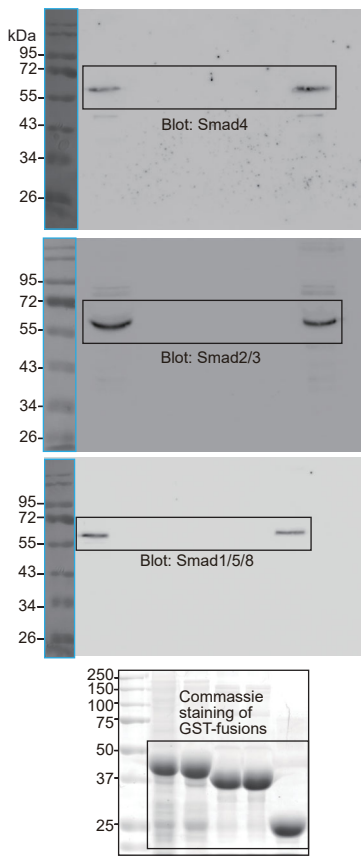

Fig. 3b

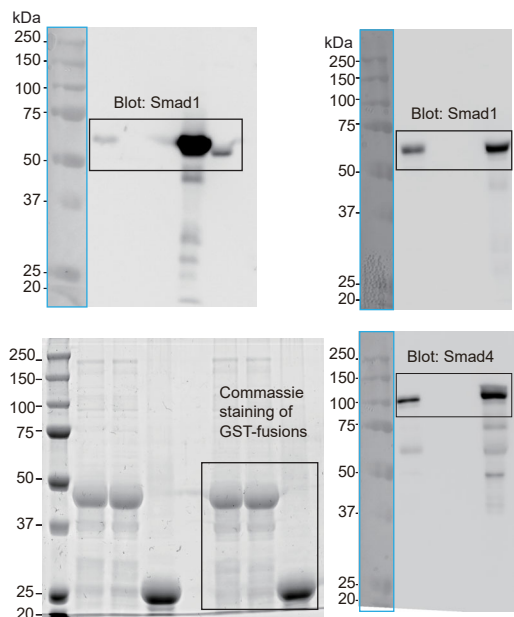

Fig. 3d

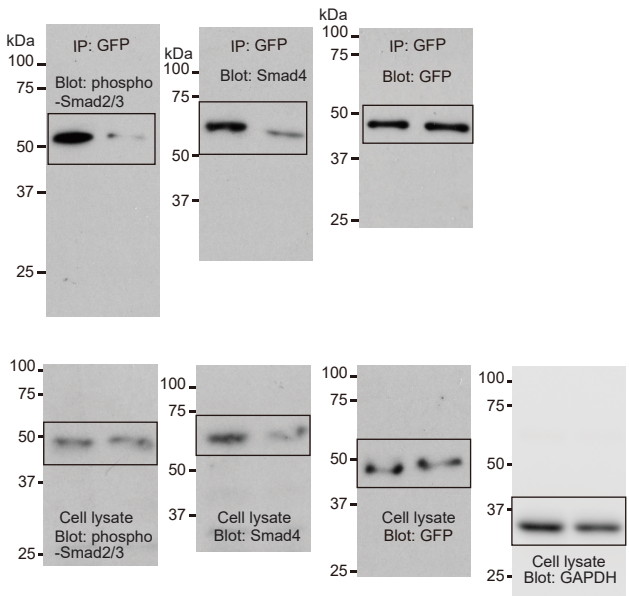

Fig. 3g

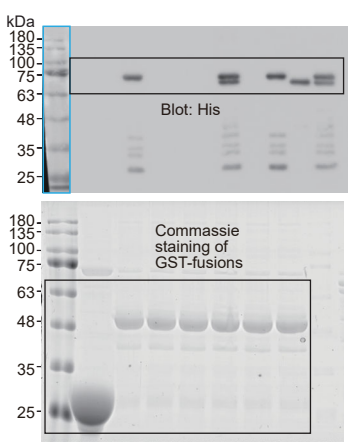

Supplement: Figure 3—source data 1. — The organization of the figure is similar to that of Figure 1—source data 1. [file elife-76146-fig3-data1.pdf]

Figure 3 - source data 2

Figure 3-  
figure supplement1a

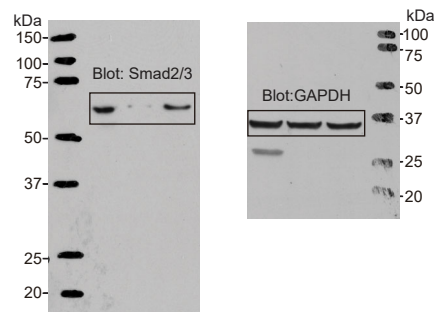

Figure 3-  
figure supplement1b

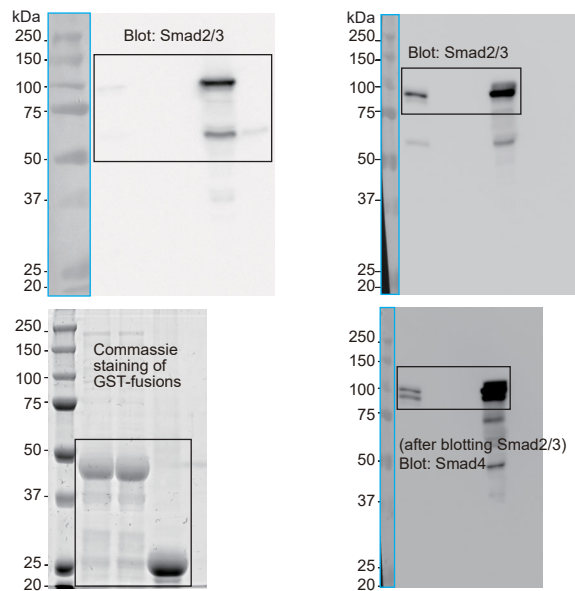

Supplement: Figure 3—figure supplement 1—source data 1. — The organization of the figure is similar to that of Figure 1—source data 1. [file elife-76146-fig3-figsupp1-data1.pdf]

Figure 4 - source data 1

Fig. 4a

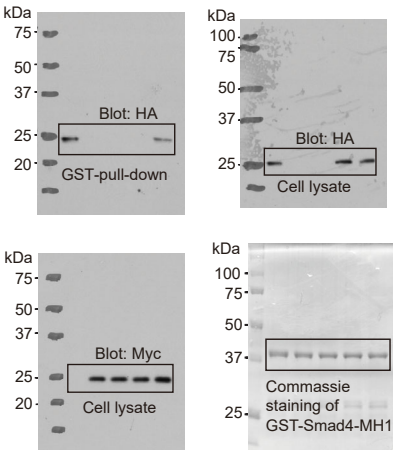

Fig. 4c

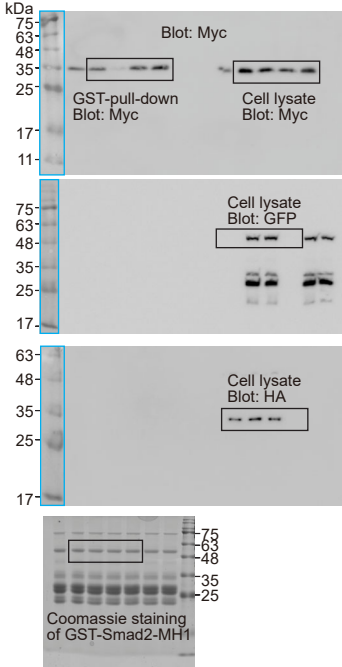

Fig. 4e

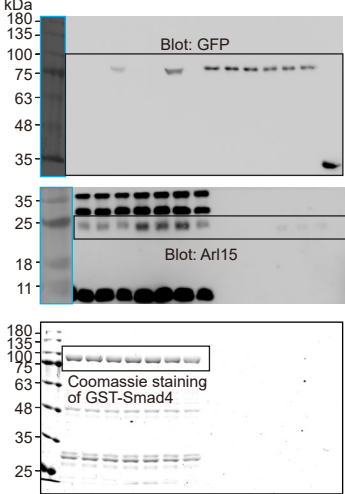

Fig. 4f

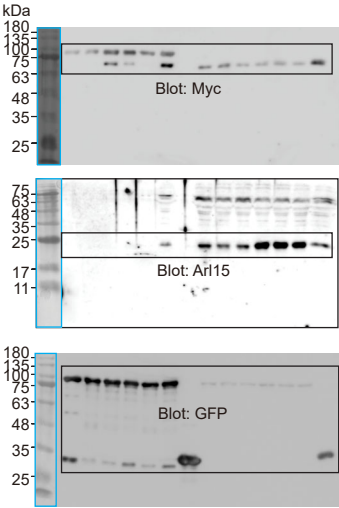

Fig. 4g

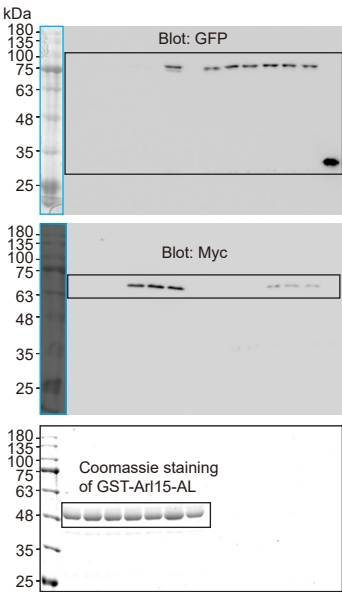

Supplement: Figure 4—source data 1. — The organization of the figure is similar to that of Figure 1—source data 1. [file elife-76146-fig4-data1.pdf]

Figure 4 - source data 2

Figure 4-  
figure supplement1

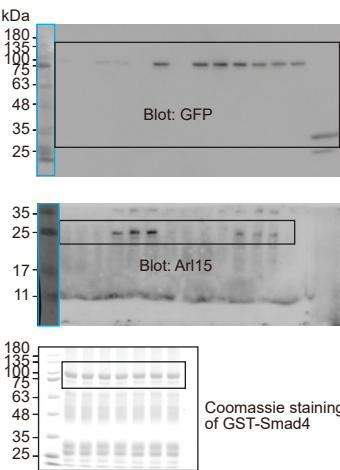

Supplement: Figure 4—figure supplement 1—source data 1. — The organization of the figure is similar to that of Figure 1—source data 1. [file elife-76146-fig4-figsupp1-data1.pdf]

Figure 5 - source data 1

Fig. 5b

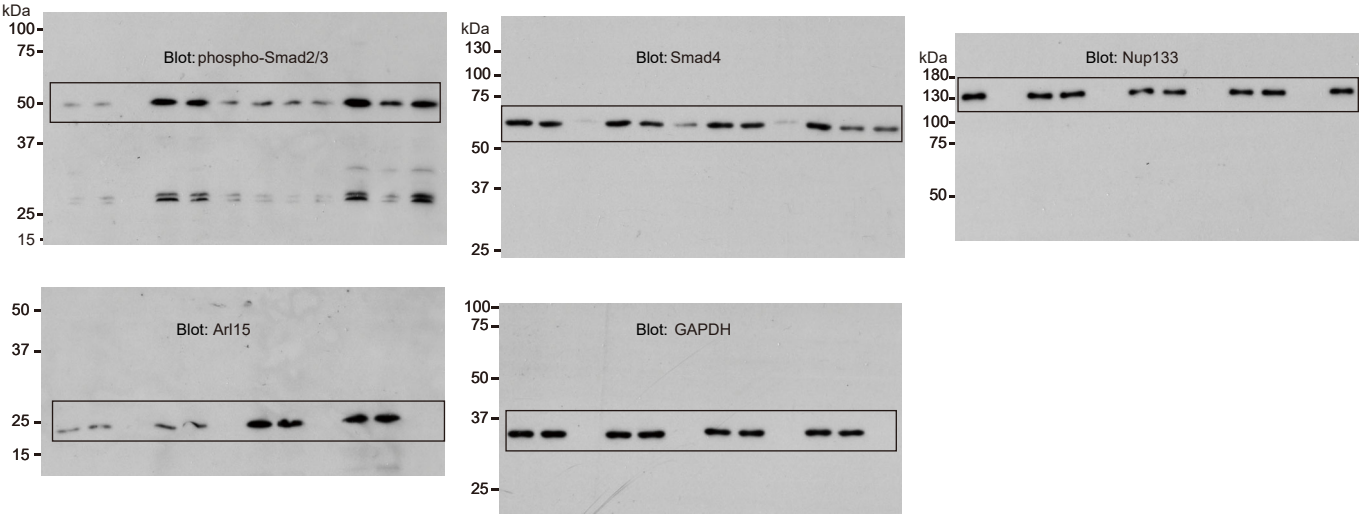

Fig. 5g

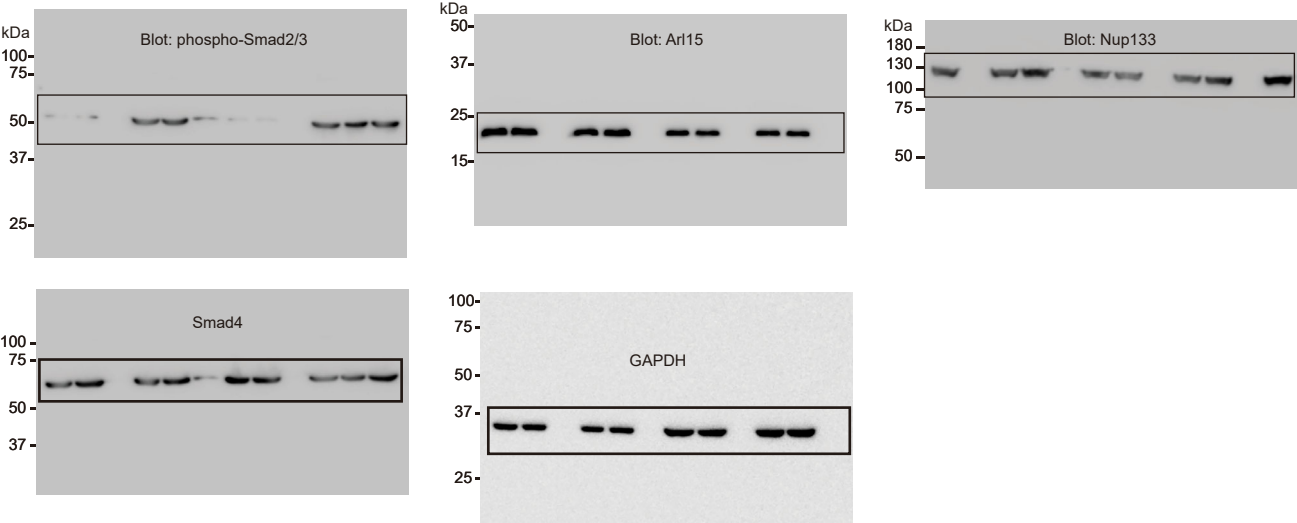

Supplement: Figure 5—source data 1. — The organization of the figure is similar to that of Figure 1—source data 1. [file elife-76146-fig5-data1.pdf]

Figure 5 - source data 2

Figure 5-  
figure supplement1

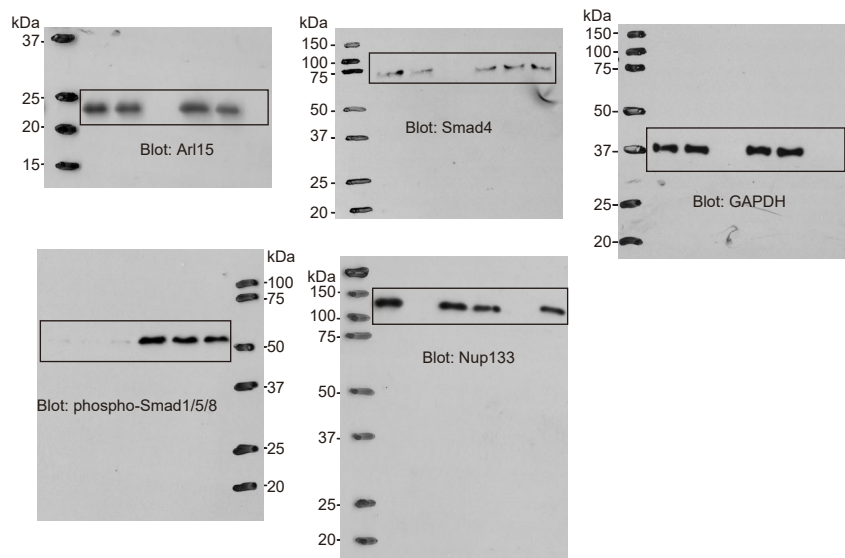

Supplement: Figure 5—figure supplement 1—source data 1. — The organization of the figure is similar to that of Figure 1—source data 1. [file elife-76146-fig5-figsupp1-data1.pdf]

Figure 7 - source data 1

Fig. 7e

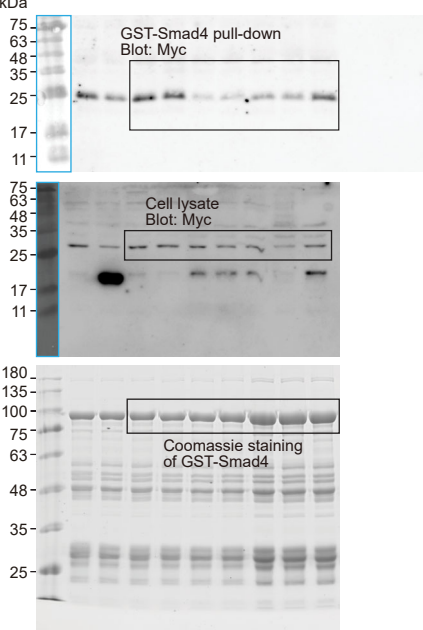

Fig. 7f

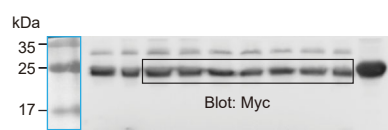

Supplement: Figure 7—source data 1. — The organization of the figure is similar to that of Figure 1—source data 1. [file elife-76146-fig7-data1.pdf]

Figure 7-  
figure supplement1

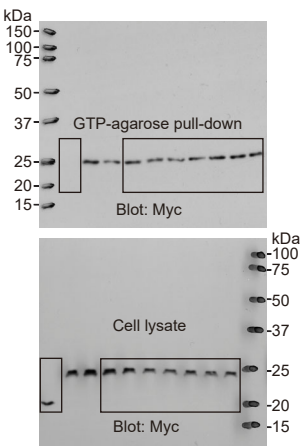

Supplement: Figure 7—figure supplement 1—source data 1. — The organization of the figure is similar to that of Figure 1—source data 1. [file elife-76146-fig7-figsupp1-data1.pdf]
