# Supplementary material for: Arl15 upregulates the TGFβ family signaling by promoting the assembly of the Smad-complex
Source: eLife. 2022 Jul 14;11:e76146. doi: 10.7554/eLife.76146 (PMC9352346; doi:10.7554/eLife.76146)
Supplement: Figure 1—source data 3. — The human kidney cDNA library (prey) was screened by Arl15-AL (bait). The table list all protein-coding clones from the screen. [file elife-76146-fig1-data3.docx]

**Figure 1 – source data 4**

List of positive hits from our yeast two-hybrid screening. The human kidney cDNA library (prey) was screened by Arl15-AL (bait). The table list all protein-coding clones from the screen.

| **No.** | **GenBank Accession No.** | **Gene Name** |
| --- | --- | --- |
| 1 | EAW62986 | Smad4 |
| 2 | NM_006793.2 | Peroxiredoxin 3 |
| 3 | NM_000146.4 | Ferritin light chain |
| 4 | NM_005697 | Secretory carrier membrane protein 2 (SCAMP 2) |
| 5 | AY430097 | DAZ associated protein 2 (DAZAP 2) |
| 6 | X02747 | Aldolase B, fructose-bisphosphate B (ALDOB) |
| 7 | NM_001101 | Actin beta (ACTB) |
| 8 | BC020807 | Proteasome 20S subunit beta 1 (PSMB1) |
| 9 | BC005109 | mRNA similar to RNA-binding protein S1, serine-rich domain |
