# Supplementary material for: Arl15 upregulates the TGFβ family signaling by promoting the assembly of the Smad-complex
Source: eLife. 2022 Jul 14;11:e76146. doi: 10.7554/eLife.76146 (PMC9352346; doi:10.7554/eLife.76146)
Supplement: Figure 1—source data 4. — Mutation information is from COSMIC. In the column ‘type of cancer identified’, the number of samples with the mutation is indicated in parenthesis. ‘Count’ displays the total number of samples with the mutation. [file elife-76146-fig1-data4.docx]

**Figure 1 – source data 5**

List of *SMAD4* missense cancer mutations that are tested in GST-Arl15-AL pull-down assay (Figure 1j and k). Mutation information is from COSMIC. In the column “type of cancer identified”, the number of samples with the mutation is indicated in parenthesis. “Count” displays the total number of samples with the mutation.

| Mutation | Type of cancer identified | Count |
| --- | --- | --- |
| M447K | Ductal carcinoma of pancreas (1) | 1 |
| D493H | Adenocarcinoma of large intestine (1), ductal carcinoma of pancreas (1), and papillary carcinoma of thyroid (1). | 3 |
| L495P | Adenocarcinoma of large intestine (1), adenocarcinoma of lung (1), and carcinoma of ovary (1). | 3 |
| R496H | Adenocarcinoma of large intestine (14), dcutal carcinoma of pancreas (3), carcinoma of endometrium (1), squamous cell carcinoma of cervix (1), and papillary carcinoma of thyroid. | 20 |
| R497H | Adenoma or adenocarcinoma of large intestine (4), carcinoma of biliary tract (1), carcinoma of lung (1), and sebaceous carcinoma of skin (1). | 7 |
| A532D | Adenocarcinoma of large intestine (2) and ductal carcinoma of pancreas (1). | 3 |
| E538K | Carcinoma of endometrium (3), transitional cell carcinoma of urinary tract (1), and adenocarcinoma of small intestine (1). | 5 |
| H541Y | Lobular carcinoma of breast (2), non-small cell carcinoma of lung (1), and carcinoma of prostate (1). | 4 |
