# Supplementary material for: Arl15 upregulates the TGFβ family signaling by promoting the assembly of the Smad-complex
Source: eLife. 2022 Jul 14;11:e76146. doi: 10.7554/eLife.76146 (PMC9352346; doi:10.7554/eLife.76146)

Figure 6 - source data 1

Figure 6-
figure supplement1c

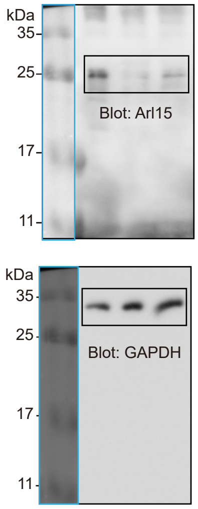

Figure 6-
figure supplement1i

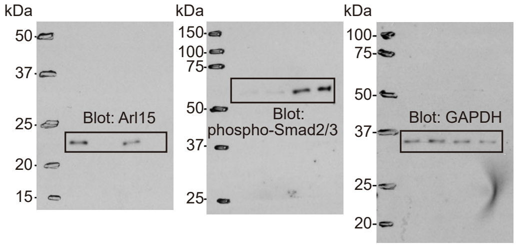

Figure 6-
figure supplement1k

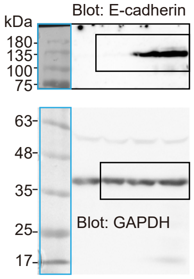

Supplement: Figure 6—figure supplement 1—source data 1. — The organization of the figure is similar to that of Figure 1—source data 1. [file elife-76146-fig6-figsupp1-data1.pdf]
