# Supplementary material for: Arl15 upregulates the TGFβ family signaling by promoting the assembly of the Smad-complex
Source: eLife. 2022 Jul 14;11:e76146. doi: 10.7554/eLife.76146 (PMC9352346; doi:10.7554/eLife.76146)
Supplement: Figure 7—source data 3. — Mutation information is from COSMIC. In the column “type of cancer identified”, the number of samples with the mutation is indicated in parenthesis. “Count” displays the total number of samples with the mutation. [file elife-76146-fig7-data3.docx]

**Figure 7 – source data 4**

List of *ARL15* missense cancer mutations that are tested in GST-Smad4 pull-down assay (Figure 7e and f). Mutation information is from COSMIC. In the column “type of cancer identified”, the number of samples with the mutation is indicated in parenthesis. “Count” displays the total number of samples with the mutation.

| Mutation | Type of cancer identified | Count |
| --- | --- | --- |
| D58N | Endometrioid carcinoma of endometrium (2). | 2 |
| E82K | Endometrioid carcinoma of endometrium (1). | 1 |
| R90L | Carcinoma of liver (1). | 1 |
| R95C | Ductal carcinoma of pancreas (2). | 2 |
| Y96F | Adenocarcinoma of lung (1). | 1 |
| R150H | Carcinoma of upper aerodigestive tract (2). | 2 |
