# Supplementary material for: Arl15 upregulates the TGFβ family signaling by promoting the assembly of the Smad-complex
Source: eLife. 2022 Jul 14;11:e76146. doi: 10.7554/eLife.76146 (PMC9352346; doi:10.7554/eLife.76146)
Supplement: Figure 7—source data 4. — In the column ‘type of cancer identified’, the number of samples with the mutation is indicated in parenthesis. ‘Count’ displays the total number of samples with the mutation. [file elife-76146-fig7-data4.docx]

**Figure 7 – source data 5**

List of *ARL15* frameshift and nonsense mutations from COSMIC. In the column “type of cancer identified”, the number of samples with the mutation is indicated in parenthesis. “Count” displays the total number of samples with the mutation.

| Mutation | Mutation type | Type of cancer identified | Count |
| --- | --- | --- | --- |
| Q74* | Nonsense | Granulosa cell tumor of ovary (1). | 1 |
| E82* | Nonsense | Adenocarcinoma of large intestine (2). | 2 |
| G84Efs*20 | Frameshift | Myoepithelial carcinoma of salivary gland (1). | 1 |
| G99* | Nonsense | Carcinoma of large intestine (1). | 1 |
| Q128* | Nonsense | Carcinoma of breast (1). | 1 |
